# A Novel Cardiac Bio-Marker: ST2: A Review

**DOI:** 10.3390/molecules181215314

**Published:** 2013-12-11

**Authors:** Marco Matteo Ciccone, Francesca Cortese, Michele Gesualdo, Roberta Riccardi, Dalia Di Nunzio, Michele Moncelli, Massimo Iacoviello, Pietro Scicchitano

**Affiliations:** Cardiovascular Diseases Section, Department of Emergency and Organ Transplantation (DETO), University of Bari, Bari 70124, Italy; E-Mails: francescacortese@hotmail.it (F.C.); michelegesualdo@libero.it (M.G.); robycikiddu@yahoo.it (R.R.); dalianoemi@alice.it (D.D.N.); michele.moncelli@hotmail.it (M.M.); massimo.iacoviello@tiscali.it (M.I.); piero.sc@hotmail.it (P.S.)

**Keywords:** ST2, heart failure, coronary artery diseases, early markers

## Abstract

Cardiovascular diseases (CVD) are the major cause of death worldwide. The identification of markers able to detect the early stages of such diseases and/or their progression is fundamental in order to adopt the best actions in order to reduce the worsening of clinical condition. Brain natriuretic peptide (BNP) and NT-proBNP are the best known markers of heart failure (HF), while troponins ameliorated the diagnosis of acute and chronic coronary artery diseases. Nevertheless, many limitations reduce their accuracy. Physicians have tried to develop further detectable molecules in order to improve the detection of the early moments of CVD and prevent their development. Soluble ST2 (suppression of tumorigenicity 2) is a blood protein confirmed to act as a decoy receptor for interleukin-33. It seems to be markedly induced in mechanically overloaded cardiac myocytes. Thus, HF onset or worsening of a previous chronic HF status, myocardial infarct able to induce scars that make the myocardium unable to stretch well, *etc*, are all conditions that could be detected by measuring blood levels of soluble ST2. The aim of this review is to explore the possible role of ST2 derived-protein as an early marker of cardiovascular diseases, above all in heart failure and ischemic heart diseases.

## 1. Introduction

Cardiovascular diseases (CVD) are the main leading cause of death worldwide [1]. Their incidence is still increasing, even in developing countries [2] and it greatly diminishes individuals’ quality of life and survival rates. 

Primary and secondary preventive actions are required in order to arrest the negative development of CVD. Thus, physicians are involved in searching for tools able to detect the early moments of CVD development. Early markers of CVD will allow immediate interventions in order to prevent the beginning of the diseases or, at least, minimize their negative consequences. Carotid intima-media thickness and brachial artery flow-mediated dilatation are instrumental, well-established early markers of atherosclerosis and chronic heart failure [3,4]. Biochemical markers work side by side with the latter ultrasonographic tools in order to detect early alterations of cardiovascular system. Ciccone *et al.* [5] recently pointed out the role of osteoprotegerin in acute/chronic coronary artery diseases (CAD). The natriuretic peptides (brain natriuretic peptide [BNP] and NT-proBNP) are the best known markers of heart failure (HF) condition [6]. Nevertheless, due to their limitations, research has been undertaken in order to implement and to find new, more accurate and complete markers of both CAD and HF conditions. Further research focused on cardiac remodelling has focused on biomechanical pathways activated during cardiac remodelling after the onset of cardiovascular diseases that could enhance production of compounds released in the bloodstream. Their early detection could detect the early moments of cardiac impairment in the case of CAD and HF. Genomic technology has discovered new genes and pathways involved in this process. A new gene, called ST2 (suppression of tumorigenicity 2) and its related protein was markedly induced in mechanically overloaded cardiac myocytes. This suggests that the protein can be overexpressed in remaining viable myocardium that has undergone great stress [7]. The aim of this review is to explore the possible role of ST2 derived-protein as an early marker of cardiovascular diseases, above all in heart failure and ischemic heart diseases.

## 2. ST2 Protein: Biological Structure and Function

Discovered in 1989, the gene named ST2 (also known as T1, IL1RL1, or Fit1) is placed on chromosome 2q12 as part of the wider interleukin 1 (IL-1) gene cluster [8]. Four isoforms are the transcriptional product of the gene and two of them are the most important: IL1RL1-b or ST2L, which is a membrane receptor member of the interleukin-1 receptor family, and IL1RL1-a or sST2a, which is a truncated soluble receptor that can be detected in serum. Alternative promoter splicing and 3' processing of the same mRNA seems to be responsible for the production of sST2 and ST2L [8]. ST2 gene shows two promoters: a proximal and a distal one, which are able to influence the mechanism of transcriptional regulation of the gene [9]. Each promoter could influence the expression of sST2 and ST2L mRNA: The alternative splicing involving the 3' end of the gene is responsible for such a difference. Baba *et al.* [9] found a different function of two GATA motifs (1 and 2), positioned upstream of the site where transcription of ST2 gene begins, which were able to influence the mRNA production and the final expression of the gene in human mast cells and in basophilic human cell line. In particular, GATA2 seemed to exert the major control in ST2 mRNA expression in both cells lines. Thus, the different expression and function of GATA motifs could play a role in the final expression and function of ST2 in immune human cells and, probably, in other cells constitutively expressing the final products of the ST2 gene transcription. Nevertheless, poor data still persists about the inner control of sST2 and ST2L expressions. Caporali *et al.* [10] found that the neurotrophin p75 receptor, a member of tumor necrosis factor (TNF)-alpha receptors family involved in post-ischemic neovascularisation in diabetes mellitus induced critical limb ischemia, is able to enhance the sST2 transcription and this action did not allow a full revascularization attempt by endothelial cells.ST2L is composed by 3 extracellular immunoglobulin G domains, a single transmembrane domain, and an intracellular domain [7,8,11]. The sST2 lacks the transmembrane and intracellular domains and it travels freely through the bloodstream.

Interleukin-33 (IL-33) has been identified as a functional ligand of ST2L [7,11]. It binds ST2L on inflammatory cells membranes: this activates mitogen-activated protein kinase (MAPK)-kinases and several biochemical pathways. The end of these reactions is the activation of the inhibitor of nuclear factor-κB (NF-κB) kinase (IKK) complex, which makes NF-kB active and able to exert its pro-inflammatory actions [8]. sST2 seems to act as a decoy-receptor for IL-33: it binds IL-33 thus subtracting such a molecule from the interaction with ST2L. sST2 interaction with IL-33 could reduce the production and activation of NF-kB, thus it would reduce inflammatory response. IL-33 has been supposed to regulate by itself the ST2L and sST2 mRNA transcription [12]. In their experimental research on highly metastatic human pancreatic adenocarcinoma cell line Colo357, Schmieder *et al.* [12] observed the influence of IL-33 and (interleukin-1) IL-1 concentrations on both ST2L and sST2 mRNA expression. The particular behavior of both cytokines lied on their skill in increasing the mRNA expression of ST2L while reducing that of sST2. When considering low doses of IL-33 mRNA expression, the presence of IL-1 was sufficient to up-regulate ST2L mRNA transcription, as well as INF-γ concentrations which rose the expression of both mRNA and ST2L protein [12]. These aspects introduce the immunomodulatory role of ST2 [13] which is more complex than previously expressed. As Sweet *et al.* [13] demonstrated, sST2 is able to directly bind and interact with macrophages when bacterial lipopolysaccharide (LPS) flows through bloodstream [13]. The interaction seems to induce an inhibition of cytokines production such as interleukin-6 (IL-6), interleukin-12 (IL-12) and TNF-alpha thus modulating inflammatory response [13]. Furthermore, ST2L seems to be able to activate T helper type 2 (Th2) lymphocytes and to induce the production of several cytokines from these cells such as IL-4, IL-5, and IL-13 which enhance Th2 response to inflammatory events [7,14].

It is interesting to note that there is a reciprocal influence between immune system cells, their production of cytokines and the final expression of ST2. IL-6 production from antigen-presenting cell is able to increase ST2 expression during Th2 differentiation [15]. Minor effects are detected for other cytokines such as IL-1, TNF-α and IL-5. Therefore, while ST2 is able to activate naive T cell to type 2 conformation, immune system is parallel able to enhance ST2 expression [15]. Nevertheless, the inner mechanisms able to explain such interactions are not fully elucidated.

## 3. The Heart and the ST2: A Tight Relationship

ST2 is involved in cardiac function and dysfunction. ST2 action in cardiac muscle is complex and not fully understood and it is tightly related to IL-33 mode of action. IL-33 could be localized simultaneously onto nuclear euchromatin and membrane-bound cytoplasmic vesicles. Kakkar *et al.* [16] demonstrated that the mechanical stretch of living cells could enhance the release of IL-33 from the cytoplasmatic vesicles. Such an exocytosis was not related to a possible stretch-induced necrosis: the cells viability was demonstrated after the periods of biaxial stretch [16]. Thus, the cellular stretch is able to increase the release of IL-33. Cardiomyocytes and cardiac fibroblasts show a parallel, increased release of both ST2L and sST2 when undergone biomechanical strain [14,17].

Such a release is an important step in the overall mechanism able to induce cardiac remodelling due to volume and/or pressure overload. IL-33 could exert an antihypertrophic effect by blocking the actions of angiotesin II or phenylephrine on myocardium. Angiotesin II or phenylephrine favour cardiac hypertrophy by inducing NF-kB action: IL-33, by interacting with ST2L, is able to attenuate NF-kB promoter activity and the phosphorylation of IKK [17]. Furthermore, it seems that the action of IL-33 via ST2L is to prevent MAPK pathway activation induced by angiotesin II or phenylephrine [17]. Histological analysis even demonstrated that ST2 presence reduced cardiac fibrosis and cardiomyocyte cross-sectional area as compared to ST2 deficient mice [17].

sST2, by acting as a decoy receptor, reduces the positive actions of IL-33 [17]. By binding IL33, it reduces the availability of the IL-33 for interaction with sarcolemmal receptor. Thus, an increase in sST2 could reduce the cardioprotective action of IL-33 on cardiac cells and could induce a negative prognostic effect on the overall cardiovascular risk profile [17]. As demonstrated by Weinberg *et al.* [14], the increased plasma levels of sST2 soon after a myocardial infarction event could be considered as a negative prognostic factor according to previous sentences.

Sanada *et al.*’s work [17] offers a more interesting point of view about the biological properties and uses of IL-33/ST2 in clinical practice. In particular, beyond its biomarker value, ST2 could be considered as a real therapeutic target. In order to demonstrate the biochemical properties of IL-33/ST2 interaction, Sanada *et al.* adopted IL-33 administration in their *in vivo*/*in vitro* experiments. It seemed that IL-33 administration was able to decrease p38 MAPK and Jun N-terminal kinase phosphorylation in MAPK pathway activation induced by angiotensin II [17]. Angiotensin II is able to exert cardiac damages by creating several reactive oxygen species (ROS): IL-33 external administration was able to reduce the ROS generation induced by angiotensin II in experimental models. Once more, an intriguing result was that the purified IL-33 2 µg/d i.p. for 4 weeks use in mice models wild type for ST2 receptor function and presence, was able to reduce the fibrotic and hypertrophic burden of cardiac cardiomyocytes and structures, as compared to ST2 −/− mice [17]. Although Sanada *et al.* concluded such part of their experiment only outlining the importance of deletion of ST2 in the general outcome of cardiac structure undergone angiotensin II action, one more idea could be represented by the possible role of ST2 as therapeutic target: IL-33 external administration in patients suffering from cardiac diseases could enhance ST2L activation and its own biochemical intracellular pathway in order to prevent or at least reduce the worsening of the clinical and structural condition of the heart. The same mice of Sanada *et al.* even demonstrated echocardiographic improvement in hypertrophy and systolic function of their heart after ST2 activation by IL-33 administration. These findings are still experimental and coming from animal models; nevertheless, the idea to develop drugs able to enhance ST2 activation in cardiac human cells could be considered as an interesting development for the next clinical future.

Nevertheless, such enthusiastic results should be tempered by novel insights about ST2 [18]. Ho *et al.* [18] pointed out the genetic landscape controlling ST2L and sST2 expression. There is a strong genetic variability among individuals about sST2 blood concentrations in basal conditions and it could account for up to 40% in inter-individual variability. The reason could be attributed to single nucleotide polymorphisms that could account for the natural variation in ST2 expression. Some variants of IL1RL1 gene (G allele of rs1558648; T allele of rs13019803) were associated with lower sST2 levels and higher mortality rate [18]. Furthermore, missense mutations in the sequence of the IL1RL1 gene regulating intracellular domain were related to peculiar behavior: these variants were able to alter the biochemical pathway downstream ST2L and this induced an up-regulation in sST2 mRNA transcription and conversion in protein. This condition was even related to higher IL-33 production [18]. Thus, the genetic variability should be taken into account when considering the role of sST2 as biomarker and/or prognostic factor; in second line, genetics should be even considered for further trial considering therapeutic actions of the IL-33/ST2L on human organs.

Thus, despite the intriguing results coming from the biological and biochemical analyses conducted, many aspects of IL-33/ST2 interaction remained unclear and deserve much more attentions and further studies. This, far from reducing the role of ST2 in clinical settings, enforces the need of more objectives evaluations in order to obtain an objective picture of the ST2 use.

## 4. sST2 Assessment: A Novel Biomarker in Cardiovascular Diseases Detection

The increased knowledge about sST2 and ST2L actions on cardiovascular system led physicians to consider the assessment of plasma levels of sST2 as a novel marker of cardiovascular events and clinical conditions, above all related to heart failure and ischemic heart diseases [19,20].

The identification of novel biomarkers able to early detect worsening signs of such clinical conditions is a fundamental step in the evolution of clinical diagnosis in order to adopt measures able to stop or at least reduce their evolution and negative outcomes. Furthermore, they could be used to detect early the benefits from therapies and the prognosis of the disease [11,21].

Nevertheless, the identification of novel molecules able to make possible a clearer and earlier overview on diseases should face the presence and the authority of previous, well-established biomarkers [20,22,23]. Troponins, natriuretic peptides, *etc*., are well-established cardiovascular diseases biomarkers already entered the international guidelines for detection of heart pathologies [24,25].

Furthermore, the optimal biomarker should be independent from other factors. The blood measurements of sST2 should not be associated to other conditions than myocardial diseases, nor linked to confounding factors that could influence its blood concentrations. This should be the “optimal condition” for a molecule in order to become a reliable biomarker. Against such a preface, one could point out the limitations of the previous “well-established” biomarkers and their dependence from clinical conditions other than the pre-established targets and, despite such limitations, they continued to be considered as important biomarkers all over the world. Why we could consider sST2 as a reliable biomarker for cardiac diseases despite its limitations will be further discussed below.

## 5. sST2 Assessment in Patients with Acute Dyspnea

sST2 has been considered as a possible biomarker in dyspnoeic patients with and without acute destabilized HF referred to the emergency department [7,11,26]. Dyspnoea is the subjective symptom of breathlessness and it often characterizes different pathologies: heart failure, chronic obstructive pulmonary disease/bronchitis/asthma, pneumonia, malignancy, pulmonary embolism, *etc*. [27]. The possibility to adopt a marker able to ameliorate diagnosis and, perhaps, differential diagnosis in dyspnoeic patients would improve their treatment and management [28,29,30]. The Pro-Brain Natriuretic Peptide Investigation of Dyspnea in the Emergency Department (PRIDE) project was a prospective, blinded study, aimed at evaluating sST2, amino-terminal pro-brain natriuretic peptide (NT-proBNP) and C-reactive protein (CRP) concentrations in 599 dyspnoeic subjects with and without acute destabilized HF admitted to the emergency department of the Massachussets General Hospital [26]. Patients supposed to show symptoms related to acute HF demonstrated sST2 serum concentrations higher than those whose symptoms seemed no-HF related (0.50 *vs.* 0.15 ng/mL; *p* < 0.001). Nevertheless, the relationship between sST2 and NT-proBNP concentrations revealed interesting results: receiver-operating characteristics (ROC) curves showed an area under the curve (AUC) of 0.74 (95% CI 0.70 to 0.78; *p* < 0.001) for sST2 when considered as a marker of acute HF [26]. The datum is inferior than that related to NT-proBNP for the same trial, *i.e.*, 0.94 (*p* < 0.0001) [31], thus the NT-proBNP confirmed its leadership as biomarker in acute HF rather than sST2. Nevertheless, sST2 seemed to have prognostic features: the combination of elevated NT-proBNP and low sST2 was observed in patients suffering from dyspnoea not related to acute HF onset [26]. Furthermore, a subgroup of patients with acute HF related symptoms showed an AUC of 0.80 (95% CI 0.75 to 0.84; *p* < 0.001) at ROC analyses regarding the relationship between sST2 and 1 year mortality [26], which was higher than the AUC value related to NT-proBNP for the same population (0.76, *p* < 0.001) [32].

Results from Socrates *et al.*’s research [29] pointed out no statistical significant difference between ROC analyses AUC regarding sST2, BNP and NT-proBNP in predicting 30 days, 90 days and 1 year mortality (30 days AUCs: sST2 = 0.76 *vs.* BNP = 0.63, *p* = 0.14; *vs.* NT-proBNP = 0.71, *p* = 0.79; 90 days AUCs: sST2 = 0.74 *vs.* BNP = 0.71, *p* = 0.5; *vs.* NT-proBNP = 0.75, *p* = 0.72. 1 year AUCs: sST2 = 0.72 *vs.* BNP = 0.71, *p* = 0.76; *vs.* NT-proBNP = 0.73, *p* = 0.68). Nevertheless, Kaplan-Meier curves showed further interesting information: the combination of sST2 to BNP and NT-proBNP measurements is able to increase the accuracy in detecting high risk dyspnoeic patients for acute HF onset. Socrates *et al.* outlined that the subgroup of patients with high sST2 levels and low levels of natriuretic peptides (both BNP/NT-proBNP) had increased mortality risk [29]. According to international guidelines [24], which do not advise sST2 evaluation in general assessment of heart failure patients, this subgroup would not receive a correct assignment. Dieplinger *et al.* [33] confirmed the same results in their recent evaluation of 251 dyspnoeic patients: sST2 is a good biomarker able to predict mortality at 1 years, above all if combined with detection of other plasma molecules (midregional-proadrenomedullin and chromogranin A).

According to Dieplinger *et al.*’s study, sST2 evaluation was not able to discriminate between patients suffering from dyspnoea due to HF or inflammatory pulmonary diseases, thus limiting its usefulness in differential diagnosis [33]. A subgroup analysis from the PRIDE [34] better evaluated such discriminatory point. The authors considered 139 patients (66% were further diagnosed with HF) who underwent complete echocardiographic evaluation at admission at the emergency department. sST2 demonstrated its tight relationship with myocardial function: both in HF and non-HF patients, sST2 was independently related to left (LV) and right (RV) ventricular function and morphological parameters at multivariate analysis (*i.e.*, RV systolic pressure: t = 2.29, *p* = 0.002; LV ejection fraction: t = −2.15, *p* = 0.05; LV end systolic dimension: t = 2.57, *p* = 0.01; LV end diastolic dimension: t = 2.98, *p* = 0.005; transmitral E to tissue Doppler Ea ratio: t = −2.13, *p* = 0.03; tissue Doppler A wave peak velocity: t = 2.11, *p* = 0.05). Shah *et al.* further demonstrated the deep impact of sST2 as a prognostic biomarker when considering a 4-year mortality. The relationship was independent from others variables: addition of log-transformed sST2 concentration as a continuous variable to other markers of clinical risk really improved the C-statistic from 0.76 to 0.80 in a statistically significant way [34].

## 6. Role of sST2 Measurements in Acute De-Compensated HF

Acute decompensated HF is the ideal clinical condition to evaluate the real burden of sST2 as biomarker and its power as prognostic factor. Boisot *et al.* enrolled 150 patients suffering from acute decompensated HF in order to evaluate the predictive 90-day mortality power of sST2 percent change as compared to well-established heart failure biomarkers such as BNP or NT-proBNP [35]. ROC analysis demonstrated the comparable predictive value of sST2 and NT-proBNP changes in predicting 90-day mortality (sST2 AUC = 0.78 CI = 0.69 to 0.88 *vs.* NT-proBNP AUC = 0.78 CI = 0.67 to 0.90) rather than BNP (AUC = 0.67 CI = 0.56 to 0.79). The optimization of the statistical analysis led physicians to consider a reduction of more than 15.5% as the best cut-off value able to identify acute decompensated HF patients at high cardiovascular risk [35]. Some authors demonstrated that a sST2 ratio (*i.e.*, the ratio between sST2 two week values and sST2 baseline values) >0.75 would be able to really predict cardiac event rates independently from others confounding factors [36]. The predictive value of sST2 still persists at 1-year follow-up: the higher the sST2 plasma levels, the higher the mortality rates at 1-year, independently of possible confounders, remaining associated to that of BNP (rs = 0.317; 95% CI, 0.157 to 0.460; *p* < 0.001) [37]. A study from Rehman *et al.* [38] revealed that CRP (T = 6.79; *p* < 0.001), body temperature (T = 5.66; *p* < 0.001), heart rate (T = 4.44; *p* < 0.001), BNP (T = 3.0; *p* = 0.003), blood urea nitrogen (T = 2.76; *p* = 0.006), smoking history (T = 2.32; *p* = 0.021), and systolic blood pressure (T = 2.01; *p* = 0.045) are all predictors of sST2 blood concentrations in acute decompensated HF [38]. Surprisingly and in agreement with the work from Manzano-Fernández *et al.* [19], left ventricular ejection fraction is not involved in prediction of sST2 blood concentrations in acute decompensated HF patients. Furthermore, Rehman *et al.* found a 72% sensitivity (95% CI: 62% to 81%), 56% specificity (95% CI: 49% to 62%), a positive predictive value of 39%, and a negative predictive value of 84% for sST2 as a predictor of mortality [38]. The combination of sST2, NT-proBNP, BNP, renal function and C-reactive protein values was able to increase the AUC for 1-year mortality prediction till 0.80 in a statistically significant manner [38]. Such results were confirmed by further studies about this matter [22] which made reliable sST2 as a real marker of acute HF and/or a good mortality predictor in the same de-compensated patients [39].

## 7. Role of sST2 Plasma Levels in Chronic HF

One of the first attempts to define the role of sST2 in chronic HF was realized in the neurohormone-sub-study of the Prospective Randomized Amlodipine Survival Evaluation 2 (PRAISE-2) study [40], *i.e.*, a multicenter, randomized, double-blinded, placebo-controlled study evaluating the effects of amlodipine 10 mg/day on survival of patients suffering from congestive HF due to non-ischemic conditions. sST2 was significantly higher in patients with severe HF (0.24 ng/mL, CI: 0.16–0.70) than controls (0.14 ng/mL, CI: 0.13–0.17; *p* < 0.0001). Scatter plots analysis suggested that sST2 values were related to BNP (r = 0.36, *p* < 0.0001), proatrial natriuretic peptide (ProANP) (r = 0.36, *p* < 0.0001), and norepinephrine (r = 0.39, *p* < 0.0001) concentrations. Nevertheless, sST2 was not able to predict mortality by itself in such a category of patients: multivariate analysis confirmed that the sST2 ratio (defined as the change in sST2 from baseline to 2 weeks treatment) was an independent predictor of mortality and/or transplantation (*p* = 0.048), above all if associated with BNP (*p* < 0.0001) and Pro-ANP (*p* < 0.0001) evaluation [40]. More interesting results came from Penn Heart Failure Study (PHFS), a multicenter, prospective cohort study involving chronic heart failure patients suffering from different range of disease severity [41]. Far from the severe HF patients of PRAISE-2, those from PHFS demonstrated that sST2 evaluation had a predictive 1-year mortality/transplantation rate similar at NT-proBNP one: the ROC curves showed comparable predictive values between the two biomarkers (sST2 AUC: 0.75, 95% CI, 0.69 to 0.79 *vs.* NT-proBNP AUC: 0.77, 95% CI, 0.72 to 0.81; *p* = 0.24) [41]. Furthermore, by combining sST2 and NT-proBNP evaluations, the AUC reached a 0.80 (95% CI, 0.76 to 0.84) value. Ky *et al.* [20] included the sST2 evaluation in a multi-markers score to be adopted in chronic HF patients in order to depict the mortality risk of such a category of patients. The new multi-markers model for chronic HF patients was superior than the Seattle Heart Failure Model (SHFM): the multi-markers score involving sST2 evaluation in combination with high-sensitivity CRP (hsCRP), myeloperoxidase (MPO), BNP, soluble fms-like tyrosine kinase receptor-1 (sFlt-1), troponin I (TnI), creatinine, and uric acid reached an AUC higher than that of SHFM (AUC multi-markers score [0.798, 95% CI 0.763–0.833 vs. AUC SHFM: 0.756, 95% CI 0.717–0.795)] at 1 year. But the combination of SHFM to the multi-markers allowed an improvement in 1-year mortality prediction up to an overall AUC of 0.803, 95% CI 0.769–0.837, *p* = 0.003.

In agreement with acute de-compensated data [19,38], Bartunek *et al.* [42] demonstrated the independence of sST2 blood levels from left ventricular systolic function. Nevertheless, chronic HF patients, above all if suffering with LV hypertrophy and/or aortic stenosis, revealed that sST2 levels were related to the diastolic overload. This reveals novel insights in the evaluation of sST2 role in myocardial function and the great importance that such a marker could play in the overall assessment of chronic HF patients [43,44]. Despite such evidences, an analysis involving 2,460 Framingham Heart Study participants (mean age 58 years) outlined that left ventricular hypertrophy and left ventricular systolic dysfunction were not related to sST2 concentrations [45]. In this recent research involving a wide sample size, sST2 failed in its predictive value, above all according to those cardiac characteristics (left ventricular hypertrophy and systolic dysfunction) that should theoretically be the main determinants of sST2 plasma levels.

## 8. sST2, IL-33 and ST2L Roles in Ischemic Heart Disease

The role of IL-33/ST2 signaling pathway in ischemic heart disease has been highlighted. *In vitro* and *in vivo* studies demonstrated that IL-33 plays a regulatory role in cardiac dysfunction after myocardial infarction [46]. In cultured rat cardiomyocytes, IL-33 treatment was dose-dependently able to inhibit hypoxia-induced apoptosis by increasing the expression of inhibitor of apoptosis proteins and by decreasing the activation of caspase-3, *i.e.*, an enzyme able to enhance apoptotic process [46]. sST2 attenuated such an effect by acting as a decoy receptor. Subcutaneous injection of IL-33 reduced infarct volume, fibrosis, and apoptosis of myocardium after infarction due to coronary ligation, while the presence of sST2 was able to counteract the positive effects of IL-33 in a dose-dependent manner [46]. IL-33/ST2 signaling pathway seems to strengthen the stability of plaques. It is well-known that interferon (IFN)-γ from Th1 lymphocytes could enhance matrix metalloproteinases (MMPs) production from macrophages [47,48]. MMPs are enzymes able to damage the fibrous cap of an atherosclerotic plaque, making it unstable [49,50]. Serum levels of MMP-9 are effectively elevated in the acute phase of myocardial infarction [51,52], showing the maximum levels of concentrations in the culprit coronary artery rather than systemic circulation [53]. IL-33 decreases serum levels of IFN-γ, thus preventing MMPs activation, extracellular matrix destruction and plaque rupture [13]. Guzel *et al.* [54] found IL-33 serum levels significantly lower in non-ST segment elevation myocardial infarction (NSTEMI) patients than controls (*p* < 0.05) and negatively correlated with MMP-9 (r = −0.461, *p* < 0.05) levels [54]. Considering an increase in sST2 [14] and a parallel decrease in IL-33 [54] levels during myocardial infarction, one can image the increased risk of patients for coronaries damages.

Shimpo *et al.* developed the predictive value of sST2 in patients suffering from acute myocardial infarction presenting in form of ST-segment elevation myocardial infarction (STEMI) [55]. Patients with higher levels of serum sST2 at STEMI presentation had a statistically significant higher risk of death or of developing new congestive heart failure at 30 days (*p* = 0.0009) [55]. Such a relationship still persisted after adjustment for confounding factors (OR: 1.77, 95% CI 1.01 to 3.12; *p* = 0.047), although the adding of BNP and cTnI made the significance loss [55]. This enforces the important role of BNP and cTnI in STEMI patients rather than sST2 blood concentrations. Considering a subgroup of the CLopidogrel as Adjunctive ReperfusIon TherapY–Thrombolysis in Myocardial Infarction 28 (CLARITY-TIMI 28) trial involving STEMI patients, Sabatine *et al.* [23] demonstrated the independent predictive value of both sST2 and NT-proBNP in a multi-markers model, with an OR of 3.57 (95% CI 1.87 to 6.81) for sST2 and 2.41 (95% CI 1.17 to 4.95) for NT-proBNP. Furthermore, the authors demonstrated that, by introducing both sST2 and NT-proBNP to the TIMI Risk Score, the c statistic of this score improved from 0.73 (95% CI, 0.68 to 0.78) to 0.78 (95% CI, 0.74 to 0.83; *p* = 0.0025) [23].

Non-ST segment elevation acute coronary syndrome (NSTE-ACS) patients did not differ from their STE-ACS counterpart [56]. sST2 plasma levels deeply increased at early stages of the event, with a peak value reached at 6–11 h. According to outcome prediction, the sST2 c-statistics for 1-year mortality was 0.67 (95% CI 0.54–0.79), which was not different from that of cTnT (0.64 [95% CI 0.53–0.76], *p* = 0.66), although lower than that of NT-proBNP one (0.78 [95% CI 0.66–0.89], *p* = 0.03). The authors further demonstrated that 1-SD increase in ln-transformed ST2 was associated with an OR 2.3 [95% CI 1.1–4.6, *p* = 0.03] in a risk model adopted within their population by considering all the clinical variable of the sample [56]. These results were similar to those from Kohli * et al.* [57] who demonstrated that high sST2 levels (*i.e.*, >35 μg/L) in NSTE-ACS patients predicted a >3-fold higher risk of cardiovascular death and heart failure at 30 days and 1 year, even after adjustment for major clinical risk factors and biomarkers (cTnI and BNP) [57]. Nevertheless, adding sST2 evaluation to GRACE score and NT-proBNP values in NSTE-ACS patients, seemed not to be long-term predictive, thus did not improve cardiovascular risk stratification [58].

Furthermore, the value of sST2 as predictor of functional recovery and remodeling of LV in the post-infarct phase was also evaluated. Weir *et al.* [59] effectively noted that sST2 serum concentrations were positively correlated with endocardial, epicardial extent and microvascular obstruction of infarct in 100 acute myocardial infarct patients. sST2 values significantly decreased from baseline to 24 weeks, predicting the degree in adverse LV functional recovery and remodeling: a slow change in sST2 concentrations was related to worse LVEF, LV end-diastolic volume index and infarct volume index [59].

## 9. Role of ST2 in Other Clinical Settings

sST2 role in predicting mortality was recently demonstrated in 588 outpatients referred for echocardiograms. The optimal cut-point of sST2 for predicting 1-year mortality was 28.25 ng/mL. Patients with high ST2 levels had an increased risk of death (*p* = 0.02); and if both ST2 and B-type natriuretic peptide levels were elevated, the risk resulted even higher (*p* = 0.01) [60].

In the Framingham Heart Study, sST2 concentrations were related to systolic blood pressure (*p* = 0.006), antihypertensive medication use (*p* = 0.03), and diabetes (*p* = 0.001) [61]. By adjusting for CAD, HF, atrial fibrillation, diabetes, hypertension, obesity, valvular disease, LV systolic dysfunction, and pulmonary and renal dysfunction, the Framingham Heart Study showed that male sex (*p* < 0.0001) and old age (*p* = 0.004) were related to higher sST2 concentrations [61].

Nevertheless, the measurement of sST2 in 348 patients presenting to the emergency department with chest pain sST2 was found to be increased in those suffering from pulmonary disease (obstructive sleep apnea, chronic obstructive pulmonary disease, asthma, pulmonary embolism, and pulmonary hypertension), systemic infection or inflammation, and alcohol abuse [62].

## 10. Conclusions

The ST2/IL-33 pathway is involved in pathophysiology of myocardial dysfunction by potentially attenuating the cardioprotective effects of IL-33. It seems to reduce the extent of cardiac damage after myocardial infarct, cardiomyocyte apoptosis, inflammatory cardiac activation, fibrosis, adverse myocardial remodeling. sST2, by acting as a decoy receptor, could prevent the beneficial effects of IL-33/ST2L interaction. sST2 measurements in blood samples could be a clinical prognostic biomarker useful in risk stratification of patients suffering from myocardial infarction, HF and dyspnea. Further studies are needed in order to better point out the evidence for a routine use of sST2 evaluation in the clinical evaluation of a patient suffering from myocardial infarction and HF.
